# Autosomal recessive limb-girdle muscular dystrophies in the Czech Republic

**DOI:** 10.1186/s12883-014-0154-7

**Published:** 2014-08-19

**Authors:** Kristýna Stehlíková, Daniela Skálová, Jana Zídková, Lenka Mrázová, Petr Vondráček, Radim Mazanec, Stanislav Voháňka, Jana Haberlová, Markéta Hermanová, Josef Zámečník, Ondřej Souček, Hana Ošlejšková, Nina Dvořáčková, Pavla Solařová, Lenka Fajkusová

**Affiliations:** 1 Centre of Molecular Biology and Gene Therapy, University Hospital Brno, Černopolní 9, Brno, 613 00, Czech Republic; 2 Central European Institute of Technology, Masaryk University, Kamenice 5, Brno, CZ-62500, Czech Republic; 3 Department of Child Neurology, University Hospital Brno, Černopolní 9, Brno, 613 00, Czech Republic; 4 Department of Neurology, Second Faculty of Medicine, Charles University and University Hospital Motol, V Úvalu 84, Prague, 150 06, Czech Republic; 5 Department of Neurology, University Hospital Brno, Jihlavská 20, Brno, 625 00, Czech Republic; 6 Department of Child Neurology, Second Faculty of Medicine, Charles University and University Hospital Motol, V Úvalu 84, Prague, 150 06, Czech Republic; 7 First Department of Pathological Anatomy, Faculty of Medicine, Masaryk University and St. Anne’s University Hospital, Pekařská 53, Brno, 656 91, Czech Republic; 8 Department of Pathology and Molecular Medicine, Second Faculty of Medicine, Charles University and University Hospital Motol, V Úvala 84, Prague, 150 06, Czech Republic; 9 Institute of Pathology, University Hospital Brno, Jihlavská 20, Brno, 625 00, Czech Republic; 10 Departmemt of Medical Genetics, University Hospital Ostrava, 17. Listopadu 1790, Ostrava, 708 52, Czech Republic; 11 Department of Medical Genetics, University Hospital Hradec Králové, Sokolská 581, 500 05 Hradec Králové, Ostrava, Czech Republic

**Keywords:** Calpain-3, CAPN3, Limb girdle muscular dystrophy, LGMD2, Sequence capture

## Abstract

**Background:**

Autosomal recessive limb-girdle muscular dystrophies (LGMD2) include a number of disorders with heterogeneous etiology that cause predominantly weakness and wasting of the shoulder and pelvic girdle muscles. In this study, we determined the frequency of LGMD subtypes within a cohort of Czech LGMD2 patients using mutational analysis of the *CAPN3, FKRP, SGCA,* and *ANO5* genes.

**Methods:**

PCR-sequencing analysis; sequence capture and targeted resequencing.

**Results:**

Mutations of the *CAPN3* gene are the most common cause of LGMD2, and mutations in this gene were identified in 71 patients in a set of 218 Czech probands with a suspicion of LGMD2. Totally, we detected 37 different mutations of which 12 have been described only in Czech LGMD2A patients. The mutation c.550delA is the most frequent among our LGMD2A probands and was detected in 47.1% of *CAPN3* mutant alleles. The frequency of particular forms of LGMD2 was 32.6% for LGMD2A (71 probands), 4.1% for LGMD2I (9 probands), 2.8% for LGMD2D (6 probands), and 1.4% for LGMD2L (3 probands).

Further, we present the first results of a new approach established in the Czech Republic for diagnosis of neuromuscular diseases: sequence capture and targeted resequencing. Using this approach, we identified patients with mutations in the *DYSF* and *SGCB* genes.

**Conclusions:**

We characterised a cohort of Czech LGMD2 patients on the basis of mutation analysis of genes associated with the most common forms of LGMD2 in the European population and subsequently compared the occurrence of particular forms of LGMD2 among countries on the basis of our results and published studies.

## Background

Limb-girdle muscular dystrophies (LGMD) are characterised by wide clinical and genetic heterogeneity. Considering this factor, achieving a precise diagnosis can be difficult and requires a comprehensive clinical and laboratory approach. LGMD are classified on the basis of an inheritance pattern and a genetic cause. Limb-girdle muscular dystrophies of type 1 (LGMD1) include forms of the disorder that have an autosomal dominant inheritance associated with a mutation in one of at least 8 genes (LGMD1A-1H). Limb-girdle muscular dystrophies of type 2 (LGMD2) include forms with an autosomal recessive inheritance and mutations in one of at least 16 genes (LGMD2A-Q).

It is helpful to take into account the geographical and ethnic origins of patients in differential diagnosis, since the relative local frequency of the different forms of LGMD varies considerably [1]–[4]. The clinical course of LGMD ranges from severe to milder forms with different age of onset and progression even within the same family [5]. Diagnosis of LGMD relies on a combination of clinical findings, results of histopathological examination of muscle biopsies (including the analysis of muscle proteins using immunohistochemistry and/or immunoblotting), followed by DNA sequencing to identify the primary mutation, which is essential for the provision of genetic and prognostic counselling.

The most frequent form of LGMD2 in Europe is probably LGMD2A, caused by mutations in the calpain-3 gene (*CAPN3*). This gene encodes a muscle-specific member of the family of Ca^2+^-activated neutral proteases that is important for muscle remodelling [6]–[8]. A marked clinical heterogeneity is observed in calpainopathies. While null-type gene mutations are usually associated with absence of calpain-3 protein in muscle and a severe phenotype, missense-type mutations are associated with unpredictable effects at both the protein and the phenotype levels [9]–[11].

Mutations in the fukutin-related protein gene (*FKRP*) result in two distinct allelic diseases: severe congenital muscular dystrophy and LGMD2I [12]. This gene encodes a putative Golgi-resident glycosyltransferase that is involved in posttranslational glycosylation of α-dystroglycan. The missense mutation c.826C > A, p.Leu276Ile is particularly common in LGMD2I patients and has been reported to confer a relatively mild phenotype when present in the homozygous state [13]–[15].

LGMD2C-2F is due to mutations in the genes encoding the components of the sarcoglycan (SG) complex. This complex, composed of 5 glycoproteins (α-, β-, γ-, δ-, ε-SG), is a member of the dystrophin-associated glycoprotein (DAG) complex localised to the sarcolemma of muscle fibres, which acts as a link between the extracellular matrix and the cytoskeleton, confers structural stability, and protects the sarcolemma from mechanical stress developed during muscle contraction. The clinical phenotype of sarcoglycanopathies ranges from a severe Duchenne-like dystrophy to a relatively mild LGMD [16]. Although the relative frequency of mutations in the different sarcoglycan genes varies from population to population, α-sarcoglycanopathy (LGMD2D) appears to be the most frequent [17].

Recently, recessive mutations in the anoctamin 5 gene (*ANO5*) were identified as a cause of LGMD2L and non-dysferlin Miyoshi myopathy [18],[19]. *ANO5* encodes a member of the anoctamin family of proteins which contains eight transmembrane domains. Dominant mutations in the *ANO5* gene are associated with the skeletal disorder gnathodiaphyseal dysplasia [20]. While the role of ANO5 is unknown, ANO1 and ANO2, which share significant sequence homology with ANO5, are known to be calcium-activated chloride channels [21]–[23]. In one study [24], analysis of *ANO5* was performed in a group of 59 British and German probands, and the mutation c.191dupA was found in 15 patients, homozygously in 11 and in compound heterozygosity with another *ANO5* variant in the rest. An intragenic SNP and an extragenic microsatellite marker were in linkage disequilibrium with the mutation, suggesting a founder effect in the populations analysed.

In this study, we determined the frequency of LGMD subtypes within a cohort of Czech LGMD2 patients using mutation analysis of the *CAPN3*, *FKRP*, *SGCA*, and *ANO5* genes. We also describe two patients with mutations in the gene encoding dysferlin (*DYSF*) and a patient with mutations in the gene encoding β-sarcoglycan (*SGCB*). These mutations were identified using Sequence capture and targeted resequencing (SeqCap-TR), a method for the capture and enrichment of selected genomic regions from full genomic DNA in a single step which, in association with targeted resequencing, allows one to focus on genomic regions of interest to discover causative mutations.

## Methods

### Patients

For analysis of genes associated with LGMD2, patients’ blood or DNA were sent to the Centre of Molecular Biology and Gene Therapy, University Hospital Brno (as the only laboratory performing LGMD genetic testing in the Czech Republic) from the Departments of Neurology and Medical Genetics of University Hospitals located in Brno, Prague, Ostrava, and Hradec Králové. Patients were included into the study on the basis of fulfilment of the following criteria: 1) a clinical phenotype characterized by progressive muscle weakness affecting primarily or predominantly the pelvic and/or shoulder girdle muscles and 2) dystrophic or myopathic features at muscle biopsy (if performed). Pathological analyses of muscle tissues were performed at the Institute of Pathology, University Hospital Brno, Brno and the Department of Pathology and Molecular Medicine, University Hospital Motol, Prague. Detailed clinical information was requested retrospectively on the basis of positive results of DNA analysis. Patients diagnosed with Duchenne muscular dystrophy, spinal muscular atrophy type III, or facioscapulohumeral muscular dystrophy were excluded using DNA analysis, and those with late-onset Pompe disease using the enzyme assay. DNA analysis reported in this study was performed with the approval of the Ethical Committee of the University Hospital Brno, and all patients or their parents gave their informed consent for participation in the study.

### Analysis of muscle tissue

Muscle tissues were routinely examined by conventional histopathological methods. Muscle protein analyses were performed using immunohistochemistry and immunoblotting as described in our previous studies [25]–[27].

### DNA analysis

Genomic DNA was extracted from peripheral blood leukocytes by the standard salting-out method and amplified. Sequences of primers for amplification of all exons and adjacent intron sequences are available on request, as well as the conditions of particular PCRs. PCR products were sequenced directly using the BigDye Terminator Cycle Sequencing Kit (Applied Biosystems) and analysed on the ABI PRISM 310 or ABI 3130xl Genetic Analyzer (Applied Biosystems). The resulting sequences were compared with the reference sequences of *CAPN3* (NCBI NG_008660.1), *FKRP* (NCBI NG_008898.1), *SGCA* (NCBI NG_008889.1), *ANO5* (NCBI NG_015844.1), *DYSF* (NCBI NG_008694.1), and *SGCB* (NCBI NG_008891.1). To find out whether the sequence variations detected had been described previously, we used the literature and the Leiden Muscular Dystrophy Pages (LMDP, http://www.dmd.nl/nmdb/home.php?action=switch_db). All novel missense mutations were screened in a control panel consisting of DNA from 150 healthy Czech individuals. The *in silico* approaches PolyPhen (http://genetics.bwh.harvard.edu/pph2/index.shtml), SIFT (http://sift.jcvi.org/www/SIFT_BLink_submit.html), and PON-P (http://structure.bmc.lu.se/PON-P2/), were used for predicting effects of novel missense mutations on the function and the structure of proteins. For prediction of effects of mutations on splicing of pre-mRNA, the *in silico* tools NetGene2 (http://www.cbs.dtu.dk/services/NetGene2/) and SpliceView (http://bioinfo4.itb.cnr.it/~webgene/wwwspliceview_ex.html) were used. The nomenclature of mutations was implemented according to the current HGVS recommendations (http://www.hgvs.org/mutnomen/). In patients with one mutation in the *CAPN3* gene, detection of deletions/duplications was performed using multiplex ligation-dependent probe amplification (MLPA). We used the SALSA MLPA P176 CAPN3 probemix kit (MRC Holland) according to the manufacturer’s guidelines. Some *CAPN3* mutations were also described at the mRNA level in our previous studies [25]–[27].

We started DNA analysis of LGMD2 patients in 2002 when sequencing analysis of the *CAPN3* gene was introduced, and results for other genes were integrated subsequently. Patients were analysed step by step for mutations in the *CAPN3, FKRP, SGCA*, and *ANO5* genes. In *CAPN3* and *SGCA*, all exons and adjacent intron regions were sequenced. In *FKRP* and *ANO5*, we analysed only exons with the occurrence of the most common mutations p.Leu276Ile and c.191dupA, respectively. In case of patients heterozygous for these mutations, analysis of all exons of a relevant gene was performed. The mentioned genes were selected on the basis of published results related to a higher incidence of associated types of LGMD2 in European populations [28]–[30]. The *CAPN3* gene was analysed in 218 Czech LGMD2 probands; the most common mutation p.Leu276Ile in the *FKRP* gene in 151 probands (negative for *CAPN3* mutations); the *SGCA* gene in 142 probands (negative for *CAPN3* and *FKRP* mutations); and the most common mutation c.191dupA in the *ANO5* gene in 136 probands (negative for *CAPN3*, *FKRP*, and *SGCA* mutations). Two patients carried the mutation p.Leu276Ile on only one *FKRP* allele and similarly 3 patients carried the mutation c.191dupA on only one *ANO5* allele. As mentioned above, analysis of all exons of the corresponding gene was performed in these cases.

### Sequence capture and targeted resequencing (SeqCap-RT)

For identification of mutations associated with neuromuscular disorders, we introduced the solution-based capture method SeqCap EZ Choice Library (Roche NimbleGene) and targeted resequencing on the GS Junior System (Roche) in 2013. A custom capture array was designed to capture exons and adjacent intron sequences of 42 genes (1020 exons, 280 kb) known to be associated with muscular dystrophies (Group 1), congenital muscular dystrophies (Group 2), congenital myopathies (Group 3), distal myopathies (Group 4), and other myopathies (Group 5), according to www.musclegenetable.fr/. The list of selected diseases and genes includes Duchenne muscular dystrophy (*DMD*), Emery-Dreifuss muscular dystrophy (*EMD*, *FHL1*, *LMNA*), limb-girdle muscular dystrophy (*MYOT*, *LMNA*, *CAV3, CAPN3*, *DYSF*, *SGCG*, *SGCA*, *SGCB*, *SGCD*, *TCAP*, *TRIM32*, *FKRP*, *TTN*, *POMT1*, *ANO5*, *FKTN*, *POMT2*, *POMGNT1*), and additionally genes associated with congenital muscular dystrophies (*LAMA2*, *LARGE*, *SEPN1*, *COL6A1*, *COL6A2*, *COL6A3*, *ITGA7*, *DNM2*), congenital myopathies, distal myopathies, and other myopathies (*NEB*, *TPM3*, *ACTA1*, *TPM2*, *TNNT1*, *CFL2*, *RYR*, *MTM1*, *BIN*, *CRYAB*, *DES*, *LAMP2*, *PABPN1*). Probes for the target regions were designed according to the “NimbleGen Sequence Capture Custom Designs” protocol. Sequence capture was performed according to the manufacturer’s instructions (NimbleGen SeqCap EZ Choice Library LR User’s Guide) with modifications of DNA fragmentation and hybridisation. We replaced nebulization by shearing using the S220 Focused Ultrasonicator (Covaris) (3 μg DNA, peak incident power 140 W, duty factor 10%, 200 cycles per burst, and treatment time 80 s) and replaced hybridization for three days by hybridization according to the NimbleGen technical note “Double Capture High Efficiency Sequence Capture of Small Targets for use in SeqCap EZ Library, Applications on 454 Sequencing Systems”. Emulsion PCR and sequencing on the GS Junior System (Roche) were performed according to the “emPCR Amplification Method Manual - Lib-L” and the “Sequencing Method Manual”. Sequencing data were evaluated using the software Sequence Pilot (JSI Medical Systems).

In the period of this study SeqCap-TR was performed in 20 probands of whom 4 had the phenotype of LGMD2 and were negative for *CAPN3*, *FKRP*, *SGCA*, and *ANO5* mutations. Here we present only results for patients in which mutations in genes associated with LGMD2 were identified, and the global results and results related to other genes are the subject of another study to be published.

## Results and discussion

Considering the wide clinical and genetic variability of LGMD, determination of particular types is a comprehensive process. Muscle biopsies still represent an important and economical step in diagnosis, but protein changes documented by immunohistochemistry can be secondary or not pronounced and therefore a definitive diagnosis requires genetic analysis. Mutations of the *CAPN3* gene are the most common cause of LGMD2, and in the set of 218 Czech probands with a suspicion of LGMD2 two mutations in this gene were identified in 67 patients. In 4 patients only one *CAPN3* mutation was determined, and sequence analysis was completed by MLPA but no gene deletions/duplications were found (Table 1). In total we identified 37 different mutations of which 12 have been described only in Czech LGMD2A patients. The most frequent mutation among our LGMD2A probands is c.550delA which was detected in 65 mutant alleles from the total number of 138 (47.1%). The patients’ clinical and pathological findings (when available) are presented in (Additional file 1: Table S1).

**Table 1:** Mutations identified in Czech LGMD2A probands

**No.**	**Mutations (cDNA level)**	**Mutations (protein level)**
1 - 23	c.550delA/c.550delA	p.(Thr184Argfs*36)/p.(Thr184Argfs*36)
24, 25	c.550delA/c.245C > T	p.(Thr184Argfs*36)/p.(Pro82Leu)
26	c.550delA**/**c.328C > T	p.(Thr184Argfs*36)/p.(Arg110*)
27	c.550delA/c.509A > G	p.(Thr184Argfs*36)/p.(Tyr170Cys)
28	c.550delA/c.598_612del	p.Thr184Argfs*36/p.Phe200_Leu204del
29	c.550delA/c.1043delG	p.(Thr184Argfs*36)/p.(Gly348Valfs*4)
30	c.550delA/c.1069C > T	p.(Thr184Argfs*36)/p.(Arg357Trp)
31	c.550delA/**c.1451T > C**	p.(Thr184Argfs*36)/**p.(Leu484Pro)**
32	c.550delA/c.1465C > T	p.(Thr184Argfs*36)/p.(Arg489Trp)
33, 34	c.550delA/c.1468C > T	p.Thr184Argfs*36/p.(Arg490Trp)
35	c.550delA/**c.1470delG**	p.(Thr184Argfs*36)/p.(Arg490Argfs*6)
36, 37	c.550delA/**c.1722delC**	p.Thr184Argfs*36/p.Ser575Leufs*20
38	c.550delA/c.1823G > A	p.(Thr184Argfs*36)/p.(Arg608Lys)
39	c.550delA/c.1981delA	p.Thr184Argfs*36/p.Ile661*
40	c.550delA/**c.2245A > C**	p.(Thr184Argfs*36)/**p.(Asn749His)**
41	**c.1A > G**/c.865C > T	**p.(Met1Val)**/p.(Arg289Trp)
42	c.133G > A/c.133G > A	p.Ala45Thr/p.Ala45Thr
43	c.146G > A/c.1069C > T	p.(Arg49His)/p.(Arg357Trp)
44	**c.224A > G/c.224A > G**	**p.Tyr75Cys/p.Tyr75Cys**
45	c.245C > T/c.245C > T	p.Pro82Leu/p.Pro82Leu
46	c.245C > T/**c.1800 + 1G > A**	p.(Pro82Leu)/**splicing**
47	c.245C > T/**c.1855C > T**	p.(Pro82Leu)/p.(Gln619*)
48	c.245C > T/c.2314_2317del	p.Pro82Leu/p.Asp772Asnfs*3
49	c.509A > G/c.509A > G	p.(Tyr170Cys)/p.(Tyr170Cys)
50	c.598_612del/c.598_612del	p.(Phe200_Leu204del)/p.(Phe200_Leu204del)
51	c.598_612del/c.640G > A	p.(Phe200_Leu204del)/p.(Gly214Ser)
52	c.598_612del/**c.2245A > C**	p.Phe200_Leu204del/**p.Asn749His**
53	c.1043delG/**c.1094G > A**	p.(Gly348Valfs*4)/p.(Trp365*)
54	c.1043delG/c.1343G > A	p.(Gly348Valfs*4)/p.(Arg448His)
55	c.1194-9A > G/**c.1800 + 1G > A**	splicing/**splicing**
56	c.1194-9A > G/c.2393C > A	splicing/p.(Ala798Glu)
57	c.1250C > T/c.1250C > T	p.(Thr417Met)/p.(Thr417Met)
58	c.1322G > A/c.1322G > A	p.Gly441Asn/p.Gly441Asn
59	c.1322delG/c.2114A > G	p.(Gly441Valfs*22)/p.(Asp705Gly)
60	c.1343G > A/**c.2093G > A**	p.(Arg448His)/**p.(Arg698His)**
61	c.1468C > T/c.2314_2317del	p.(Arg490Trp)/p.(Asp772Asnfs*3)
62	**c.1788_1793del**/c.2242C > T	**p.Lys597_Lys598del**/p.Arg748*
63 - 66	c.598_612del/c.1746-20C > G	p.(Phe200_Leu204del)/splicing
67	**c.614T > C**/c.1746-20C > G	**p.(Leu205Pro)**/splicing
68, 69	c.550delA/-	p.(Thr184Argfs*36)/-
70, 71	c.598_612del/-	p.(Phe200_Leu204del)/-

Mutations in bold letters were detected only in Czech LGMD2A patients.

Probands negative for *CAPN3* mutations (151) were analysed for the most common mutation in the *FKRP* gene, p.Leu276Ile. The homozygous occurrence of this mutation was identified in 7 patients. In two patients heterozygous for p.(Leu276Ile), we were able to identify a second mutation: patient 79 carries the mutation p.(Pro316Arg) described in [12] and patient 80 has the new mutation p.(Trp359Ser) (Table 2).

**Table 2:** Mutations identified in Czech LGMD2I, 2D, 2 L, 2B, and 2E probands

**No.**	**Gene**	**Mutation (cDNA level)**	**Mutation (protein level)**
72 - 78	*FKRP*	c.826C > A/c.826C > A	p.(Leu276Ile)/p.(Leu276Ile)
79	*FKRP*	c.826C > A/c.947C > G	p.(Leu276Ile)/p.(Pro316Arg)
80	*FKRP*	c.826C > A/**c.1076G > C**	p.(Leu276Ile)/**p.(Trp359Ser)**
81	*SGCA*	c.229C > T/c.229C > T	p.(Arg77Cys)/p.(Arg77Cys)
82, 83	*SGCA*	c.157 + 1G > A/c.850C > T	splicing/p.(Arg284Cys)
84	*SGCA*	c.229C > T/c.739G > A	p.(Arg77Cys)/p.(Val247Met)
85	*SGCA*	c.290A > G**/c.303dupA**	p.(Asp97Gly)/p.(Gln101Glnfs*4)
86	*SGCA*	c.229C > T/c.308 T > C	p.(Arg77Cys)/p .(Ile103Thr)
87	*ANO5*	c.191dupA/*c.966A > T*	p.(Asn64Lysfs*15)/*p.(Leu322Phe)*
88, 89	*ANO5*	c.191dupA/c.2272C > T	p.(Asn64Lysfs*15)/p.(Arg758Cys)
90	*DYSF*	c.3832C > T/**c.5509G > T**	p.(Gln1278*)/**p.(Asp1837Tyr)**
91	*DYSF*	c.509C > A/**c.5907G > C**/c.610C > T/c.1120G > C/	p.(Ala170Glu)/**p.(Trp1969Cys)**/p.(Arg204*)/p.(Val374Leu)/
92	*SGCB*	c.341C > T/c.341C > T	p.(Ser114Phe)/p.(Ser114Phe)

Mutations in bold letters were detected only in Czech LGMD2 patients. The variant c.966A > T in italics is probably a nucleotide polymorphism (LMDP, dbSNP-rs7481951).

In 142 probands without mutations in the *CAPN3* or *FKRP* genes, we performed analysis of the *SGCA* gene. Mutations were detected in 6 patients (Table 2). The most common mutation among our LGMD2D patients is p.(Arg77Cys), which was present in 4 mutant alleles. We also identified one new *SGCA* mutation, c.303dupA (patient 85).

Probands negative for *CAPN3*, *FKRP*, and *SGCA* mutations (136) were screened for the most common mutation in the *ANO5* gene, c.191dupA. This mutation was found heterozygously in 3 patients, and subsequent sequencing analysis of all exons and adjacent intron regions detected the mutation p.(Arg758Lys) in two of them (Table 2). This mutation was described in combination with c.191dupA in other studies [24], [31] and the associated phenotypes corresponded to distal non-dysferlin Miyoshi myopathy, unlike our patient 88 whose phenotype matches rather LGMD2L (a detailed description of the phenotype of patient 89 was not available). Analysis for this mutation was subsequently performed in all LGMD2 patients with unconfirmed diagnosis at the DNA level, but it was not detected. In patient 87 carrying c.191dupA on one allele, we did not identify a second mutation but only the polymorphism p.(Leu322Phe) described in LMDP. The patient’s clinical and pathological findings are presented in (Additional file 2: Table S2).

The aim of this study was to evaluate the relative proportion of the most frequent types of LGMD2 identified in other European countries in Czech probands with a suspicion of LGMD2. The results show that the frequency of the forms of LGMD2 which were analysed is 32.6% for LGMD2A (71 probands), 4.1% for LGMD2I (9 probands), 2.8% for LGMD2D (6 probands), and 1.4% for LGMD2L (3 probands). These results indicate that there is good agreement between the frequency of particular forms of LGMD2 in the Czech Republic and in Italy (respectively LGMD2A 31.1%, LGMD2I 7.4%, LGMD2D 8.4%, LGMD2L 2.1% [28] and LGMD2A 28.4%, LGMD2I 6.4%, LGMD2D 8.3% [2]), in contrast to studies from Denmark where the frequency of LGMD2A is significantly lower (12.1%) and that of LGMD2I significantly higher (38.4%) [29] (Table 3).

**Table 3:** Frequency of LGMD2A, 2I, 2D, 2 L in European countries

**Litera-ture**	**Population**	**Number of patients analysed in the study**	**Number and percentage of patients with LGMD2A**	**Number and percentage of patients with LGMD2I**	**Number and percentage of patients with LGMD2D**	**Number and percentage of patients with LGMD2L**
This study	Czech	218 LGMD2 probands	71 (67 had two mutations, 4 one); **32.6%**	9; **4.1%**	6; **2.8%**	3; **1.4%**
[28]	Italy	190 LGMD probands	59; **31.1%**	14; **7.4%**	16; **8.4%**	4; **2.1%**
[2]	Italy	155 LGMD probands	44 (30 had two mutations, 14 one); **28.4%**	10; **6.4%**	13; **8.4%**	NI
[32]	Italy	550 patients with LGMD, myopathy, or asymptomatic hyperCKemia	102; **18.5%**	16; **2.8%**	37; **6.7%**	NI
[33]	Italy	519 patients with LGMD, myopathy, or asymptomatic hyperCKemia	94 (76 had two mutations, 18 one); **18.1%**	NI	NI	NI
[10]	Italy	530 patients with muscular dytrophy	141 (104 had two mutations and 37 one); **26.6%**	NI	NI	NI
[34]	Italy	214 probands with muscular dystrophy or myopathy	NI	13 (9 had two mutations, 4 one); **6.1%**	NI	NI
[35]	Germany	98 probands with LGMD2 and 102 probands with asymptomatic or minimally symptomatic hyperCKemia	NI	7; **3.5%**	NI	NI
[29]	Denmark	99 LGMD2 patients	12; **12.1%**	38; **38.4%**	NI	NI
[4]	Dutch	67 LGMD probands	14; **20.9%**	5; **7.5%**	NI	NI
[30]	Dutch	68 LGMD probands	NI	NI	NI	11; **16.2%**

NI: no information.

Histopathological and immunohistochemical analysis of muscle tissue performed in 42 of the 71 patients with identified mutation(s) in the *CAPN3* gene showed that 34 patients had a dystrophic and 8 had a myopathic pattern of muscle tissue. Immunoblotting of the CAPN3 protein was implemented in 33 patients. In patients with a mutation creating a protein termination codon (PTC) on both *CAPN3* alleles, immunoblotting showed the absence of 94 kDa CAPN3 (16 cases); in patients with a combination PTC/missense mutation we noted the absence (4 patients) or weak labelling (2 patients) of CAPN3; and in cases with a combination missense/missense mutation we detected the absence (2 patients), weak labelling (1 patient), or normal labelling (1 patient) of CAPN3. Other cases encompassing combinations of in-frame deletions, splicing mutations, or with a mutation on one *CAPN3* allele occurred exceptionally (Additional file 1).

Analysis of muscle tissue performed in 6 patients with mutations in the *FKRP* gene showed a dystrophic pattern of muscle tissue. In two cases (patients 72 and 78), immunohistochemical analysis of alpha-dystroglycan was performed and both patients had a deficit of this protein. Three patients with mutations in the *SGCA* gene showed absence or weak labelling of alpha, beta, and gamma-sarcoglycans (patients 82, 85), but in contrast immunodetection of these proteins was normal in patient 84 (Additional file 2).

In 2013, we introduced the SeqCap-TR method for genetic diagnosis of neuromuscular disorders and this approach was applied in 20 patients, 4 of whom had the LGMD2 phenotype and were negative for *CAPN3*, *FKRP*, *SGCA*, and *ANO5* mutations. We identified two patients with LGMD2B (mutations in the *DYSF* gene), one patient with LGMD2E (mutations in the *SGCB* gene), and one patient is without identified causal mutations as yet. Patient 90 is a compound heterozygote for two *DYSF* mutations (genotype p.[Gln1278*]; [Asp1837Tyr]). Compound heterozygous and homozygous occurrence of p.(Gln1278*) was described in [36], in the case of a homozygous patient an initial phenotype corresponded to Miyoshi myopathy. The mutation c.5509G > T, p.(Asp1837Tyr) was not reported so far, but c.5509G > A, p.(Asp1837Asn) was described in several studies. In [37] a patient carried the genotype p.[Asp1837Asn]; [Trp1968*] and the phenotype of LGMD2B, and in [38] and [39] patients with the genotype p.[Asp1837Asn]; [Tyr522*] had Miyoshi myopathy. In cases of homozygous occurrence of this mutation, phenotypes of Miyoshi myopathy [40]–[42] and also of LGMD2B [42] were observed. The phenotype of our patient 90 corresponded rather with LGMD2B. Patient 91 carries 4 mutations in the *DYSF* gene, p.(Ala170Glu), p.(Arg204*), p.(Val374Leu), and the novel mutation p.(Trp1969Cys). The mutation p.(Ala170Glu) is described in the LMDP as unknown pathogenic, probably pathogenic, or pathogenic and p.(Val374Leu) as probably not pathogenic or pathogenic. The evaluation of these missense mutations using *in silico* tools is described in (Additional file 3: Table S3) together with the evaluation of selected missense mutations identified in the genes analysed. DNA analysis was performed in patient’s parents; her father carries p.(Arg204*) and p.(Val374Leu), and her mother carries p.(Ala170Glu) and p.(Trp1969Cys). On the basis of the *in silico* analyses and inheritance in the patient’s family, we suppose that p.(Arg204*) and p.(Trp1969Cys) are causal mutations. Patient 92 is homozygous for the *SGCB* mutation p.(Ser114Phe) described in the LMDP in association with LGMD2E. The patients’ clinical and pathological findings are shown in (Additional file 2: Table S2). None of the novel missense mutations were detected in 150 control DNA samples.

## Conclusions

We characterised a cohort of Czech LGMD2 patients on the basis of analysis of mutations in genes associated with the most common forms of LGMD2 in the European population and compared these results with published studies. This study expands our previous results for the Czech LGMD2A population [25]–[27], as well as the spectrum of mutations in other types of LGMD2, and further provides information concerning clinical and genetic correlations. At the present time, molecular genetic diagnosis of Czech LGMD2 patients follows the following scheme: sequencing of all exons and adjacent intron regions of the *CAPN3* gene, detection of the most common mutation p.Leu276Ile in the *FKRP* gene, and sequence capture and targeted resequencing of genes associated with neuromuscular disorders.

## Abbreviations

DAG complex: Dystrophin-associated glycoprotein complex

LGMD: Limb-girdle muscular dystrophy

LMDP: Leiden muscular dystrophy pages

MLPA: Multiplex ligation-dependent probe amplification

SG complex: Sarcoglycan complex

SeqCap-TR: Sequence capture and targeted resequencing

## Competing interests

The authors declare that they have no competing interests.

## Authors’ contributions

KS, DS, JZ carried out the molecular genetic studies. LM, PV, RM, SV, JH, HO, ND, PS performed clinical diagnostics of LGMD patients. MH, JZ, OS were involved in pathological analysis of muscle tissue. LF participated in design study and manuscript preparation. All authors read and approved the final manuscript.

## Additional files

## Supplementary Material

Additional file 1: Table S1. Mutations and pathological-clinical findings identified in Czech LGMD2A probands. Click here for file

Additional file 2: Table S2. Mutations and pathological-clinical findings identified in Czech LGMD2I, 2D, 2L, 2B, and 2E probands. Click here for file

Additional file 3: Table S3. *In silico* prediction of effects of selected missense mutations. Click here for file
