# Supplementary material for: Autosomal recessive limb-girdle muscular dystrophies in the Czech Republic
Source: BMC Neurol. 2014 Aug 19;14:154. doi: 10.1186/s12883-014-0154-7 (PMC4145250; doi:10.1186/s12883-014-0154-7)
Supplement: Additional file 1: Table S1. — Mutations and pathological-clinical findings identified in Czech LGMD2A probands. [file s12883-014-0154-7-S1.docx]

Table S1. Mutations and pathological-clinical findings identified in Czech LGMD2A probands

| No. | **Mutations (cDNA level)** | **Mutations (protein level)** | **Histochemistry; immunohistochemistry; Western blot of CAPN3** | **Onset (years)** | **First manifestation of muscle weakness** | **Age (years)** | **Actual localisation of muscle weakness** | **Loss of walking (years)** | **Contrac-tures** | **CK (μkat/l)** | **Other symptoms** |
| --- | --- | --- | --- | --- | --- | --- | --- | --- | --- | --- | --- |
| 1 | c.550delA/ c.550delA | p.(Thr184Argfs*36)/ p.(Thr184Argfs*36) | Dystrophic pattern; normal immunolabelling; CAPN3: absence on 94, 60, 30 kDa, labelling on 45 kDa | 6 | Pelvic-femoral girdles | 32 | Shoulder and pelvic-femoral girdles | 22 | Achilles tendons | NI | Hyperlordosis, respiratory muscle weakness |
| 2 | c.550delA/ c.550delA | p.(Thr184Argfs*36)/ p.(Thr184Argfs*36) | Dystrophic pattern; normal immunolabelling; CAPN3: absence on 94, 60, 30 kDa, labelling on 45 kDa | 12 | Shoulder and pelvic-femoral girdles | 34 | Shoulder and pelvic-femoral girdles, distal muscles | 30 | Achilles tendons | 88 | no |
| 3 | c.550delA/ c.550delA | p.(Thr184Argfs*36)/ p.(Thr184Argfs*36) | NP | 15 | Pelvic-femoral girdles | 16 | Pelvic-femoral girdles | no | no | 83 | Calf hypertrophy |
| 4 | c.550delA/ c.550delA | p.(Thr184Argfs*36)/ p.(Thr184Argfs*36) | NP | 15 | Pelvic-femoral girdles | 34 | Pelvic-femoral girdles, LL distal muscles | 32 | no | 15 | no |
| 5 | c.550delA/ c.550delA | p.(Thr184Argfs*36)/ p.(Thr184Argfs*36) | No muscle fibres | 8 | Pelvic-femoral girdles | 35 | Shoulder and pelvic-femoral girdles | no | Achilles tendons | 10 | Hyperlordosis, muscle stiffness (after rest) |
| 6 | c.550delA/ c.550delA | p.(Thr184Argfs*36)/ p.(Thr184Argfs*36) | NP | 11 | Pelvic-femoral girdles | 14 | Pelvic-femoral girdles | no | no | 140 | no |
| 7 | c.550delA/ c.550delA | p.(Thr184Argfs*36)/ p.(Thr184Argfs*36) | Dystrophic pattern; immunolabelling: dysferlin deficiency; CAPN3: absence on 94, 60, 30 kDa, labelling on 45 kDa | 6 | Pelvic-femoral girdles | 10 | Pelvic-femoral girdles | no | Achilles tendons | 100 | Musle pain |
| 8 | c.550delA/ c.550delA | p.(Thr184Argfs*36)/ p.(Thr184Argfs*36) | NP | 8 | Pelvic-femoral girdles, muscle fatigue | 31 | Shoulder and pelvic-femoral girdles | Weelchair bound for longer distance | Achilles tendons | 5 | Hyperlordosis |
| 9 | c.550delA/ c.550delA | p.(Thr184Argfs*36)/ p.(Thr184Argfs*36) | NP | 20 | Pelvic-femoral girdles | 35 | Shoulder and pelvic-femoral girdles, distal muscles | 35 | Achilles tendons, elbows, wrists, fingers | 2 | no |
| 10 | c.550delA/ c.550delA | p.(Thr184Argfs*36)/ p.(Thr184Argfs*36) | Dystrophic pattern; immunolabelling: dysferlin deficiency; CAPN3: absence on 94, 60, 30 kDa, labelling on 45 kDa | 8 | Pelvic-femoral girdles | 22 | Pelvic-femoral girdles, LL distal muscles | no | no | 68 | no |
| 11 | c.550delA/ c.550delA | p.(Thr184Argfs*36)/ p.(Thr184Argfs*36) | Dystrophic and inflammatory pattern; normal immunolabelling; CAPN3: NP | 14 | Shoulder and pelvic-femoral girdles | 15 | Shoulder and pelvic-femoral girdles | no | Achilles tendons | 90 | Pain of calves |
| 12 | c.550delA/ c.550delA | p.(Thr184Argfs*36)/ p.(Thr184Argfs*36) | Dystrophic pattern; immunolabelling: dysferlin deficiency; CAPN3: absence on 94 and 30 kDa, weak labelling on 60 kDa, labelling on 45 kDa | 6 | Shoulder and pelvic-femoral girdles | 7 | Shoulder and pelvic-femoral girdles | no | Achilles tendons, elbows, wrists, fingers | 111 | no |
| 13 | c.550delA/ c.550delA | p.(Thr184Argfs*36)/ p.(Thr184Argfs*36) | Dystrophic pattern; immunolabelling: dysferlin deficiency; CAPN3: absence on 94, 60, 30 kDa, labelling on 45 kDa | 16 | Pelvic-femoral girdles | 18 | Pelvic-femoral girdles | no | no | 58 | Muscle cramps |
| 14 | c.550delA/ c.550delA | p.(Thr184Argfs*36)/ p.(Thr184Argfs*36) | NP | 19 | Pelvic-femoral girdles | 30 | Shoulder and pelvic-femoral girdles | no | no | 14 | no |
| 15 | c.550delA/ c.550delA | p.(Thr184Argfs*36)/ p.(Thr184Argfs*36) | Dystrophic pattern; normal immunolabelling; CAPN3: absence on 94, 60, 30 kDa, labelling on 45 kDa | 8 | Pelvic-femoral girdles | 10 | Pelvic-femoral girdles, LL distal muscles | no | no | 86 | no |
| 16 | c.550delA/ c.550delA | p.(Thr184Argfs*36)/ p.(Thr184Argfs*36) | Dystrophic pattern; immunolabelling: dysferlin deficiency; CAPN3: absence on 94, 60, 30 kDa, labelling on 45 kDa | 5 | Pelvic-femoral girdles | 27 | Shoulder and pelvic-femoral girdles, abdominal muscles | 19 | Achilles tendons, elbows, wrists, fingers | 4 | no |
| 17 | c.550delA/ c.550delA | p.(Thr184Argfs*36)/ p.(Thr184Argfs*36) | Dystrophic pattern; immunolabelling: dysferlin deficiency; CAPN3: absence on 94, 60, 30 kDa, labelling on 45 kDa | 10 | Shoulder and pelvic-femoral girdles | 32 | Shoulder and pelvic-femoral girdles | 30 | Achilles tendons | 92 | no |
| 18 | c.550delA/ c.550delA | p.(Thr184Argfs*36)/ p.(Thr184Argfs*36) | Dystrophic pattern; normal immunolabelling; CAPN3: absence on 94, 60, 30 kDa, labelling on 45 kDa | 14 | Pelvic-femoral girdles | 14 | Pelvic-femoral girdles | no | Achiles tendons | 95 | Stiffness and pain of calves, scapula alata |
| 19 | c.550delA/ c.550delA | p.(Thr184Argfs*36)/ p.(Thr184Argfs*36) | Dystrophic pattern; normal immunolabelling; CAPN3: absence on 94, 60, 30 kDa, labelling on 45 kDa | NI | NI | 20 | Shoulder and pelvic-femoral girdles | NI | NI | NI | NI |
| 20 | c.550delA/ c.550delA | p.(Thr184Argfs*36)/ p.(Thr184Argfs*36) | NP | NI | NI | 34 | Shoulder and pelvic-femoral girdles | NI | NI | NI | NI |
| 21 | c.550delA/ c.550delA | p.(Thr184Argfs*36)/ p.(Thr184Argfs*36) | NP | NI | NI | 38 | Shoulder and pelvic-femoral girdles | NI | NI | NI | NI |
| 22 | c.550delA/ c.550delA | p.(Thr184Argfs*36)/ p.(Thr184Argfs*36) | NP | NI | NI | 23 | Shoulder and pelvic-femoral girdles | NI | NI | NI | NI |
| 23 | c.550delA/ c.550delA | p.(Thr184Argfs*36)/ p.(Thr184Argfs*36) | NP | NI | NI | 37 | Shoulder and pelvic-femoral girdles | NI | NI | NI | NI |
| 24 | c.550delA/ c.245C>T | p.Thr184Argfs*36/ p.Pro82Leu | Dystrophic pattern; normal immunolabelling; CAPN3: absence on 94 and 30 kDa, labelling on 60 kDa | 12 | Shoulder and pelvic-femoral girdles | 28 | Shoulder and pelvic-femoral girdles, distal muscles | no | Achilles tendons | 62 | no |
| 25 | c.550delA/ c.245C>T | p.(Thr184Argfs*36)/ p.(Pro82Leu) | Dystrophic pattern; immunolabelling: dysferlin deficiency; CAPN3: weak labelling on 94 kDa and 45 kDa, absence on 60 and 30 kDa | 10 | Shoulder and pelvic-femoral girdles | 31 | Shoulder and pelvic-femoral girdles, distal muscles | no | Achilles tendons | 85 | no |
| 26 | c.550delA**/** c.328C>T | p.(Thr184Argfs*36)/ p.(Arg110*) | Dystrophic pattern; normal immunolabelling; CAPN3: absence on 94, 60, 30 kDa, labelling on 45 kDa | NI | NI | 27 | Shoulder and pelvic-femoral girdles | NI | NI | NI | NI |
| 27 | c.550delA/ c.509A>G | p.(Thr184Argfs*36)/ p.(Tyr170Cys) | NP | 9 | Shoulder and pelvic-femoral girdles | 10 | Shoulder and pelvic-femoral girdles | no | Achilles tendons | 42 | no |
| 28 | c.550delA/ c.598_612del | p.Thr184Argfs*36/ p.Phe200_Leu204del | NP | 5 | Pelvic-femoral girdles | 13 | Pelvic-femoral girdles, LL distal muscles | no | no | 63 | no |
| 29 | c.550delA/ c.1043delG | p.(Thr184Argfs*36)/ p.(Gly348Valfs*4) | Dystrophic and inflammatory pattern; normal immunolabelling; CAPN3: absence on 94, 60, 30 kDa, labelling on 45 kDa | 10 | Shoulder and pelvic-femoral girdles | 21 | Shoulder and pelvic-femoral girdles, distal muscles | 20 | no | 76 | no |
| 30 | c.550delA/ c.1069C>T | p.(Thr184Argfs*36)/ p.(Arg357Trp) | NP | 16 | Pelvic-femoral girdles | 20 | Pelvic-femoral girdles | no | no | 90 | no |
| 31 | c.550delA/ **c.1451T>C** | p.(Thr184Argfs*36)/ **p.(Leu484Pro)** | NP | 16 | Shoulder and pelvic-femoral girdles | 59 | Shoulder and pelvic-femoral girdles, distal muscles | 50 | Achilles tendons, knees | 48 | no |
| 32 | c.550delA/ c.1465C>T | p.(Thr184Argfs*36)/ p.(Arg489Trp) | Dystrophic and inflammatory pattern, intersticium with presence of numerous eosinophils (eosinophilic myositis was suspected); normal immunolabelling; CAPN3: NP | 12 | Pelvic-femoral girdles | 15 | Pelvic-femoral girdles | no | no | 70 | no |
| 33 | c.550delA/ c.1468C>T | p.Thr184Argfs*36/ p.Arg490Trp | Dystrophic pattern; immunolabelling: focal dysferlin deficiency; CAPN3: NP | 8 | Shoulder and pelvic-femoral girdles | 30 | Shoulder and pelvic-femoral girdles, distal muscles | no | Achilles tendons | 62 | no |
| 34 | c.550delA/ c.1469G>A | p.(Thr184Argfs*36)/ p.(Arg490Gln) | NP | 26 | Shoulder and pelvic-femoral girdles | 56 | Shoulder and pelvic-femoral girdles | 56 | no | 3 | no |
| 35 | c.550delA/ **c.1470delG** | p.(Thr184Argfs*36)/ **p.(Arg490Argfs*6)** | NP | NI | NI | 16 | Pelvic-femoral girdles | NI | NI | NI | NI |
| 36 | c.550delA/ **c.1722delC** | p.Thr184Argfs*36/ **p.Ser575Leufs*20** | Myopathic pattern; normal immunolabelling; CAPN3: absence on 94, 60, 30 kDa, labelling on 45 kDa | 10 | Pelvic-femoral girdles | 13 | Pelvic-femoral girdles | no | Achille tendons | 74 | Calf hypertrophy, hyperlordosis |
| 37 | c.550delA/ **c.1722delC** | p.Thr184Argfs*36/ **p.Ser575Leufs*20** | Myopathic pattern; normal immunolabelling; CAPN3: absence on 94, 60, 30 kDa, labelling on 45 kDa | 7 | Pelvic-femoral girdles | 9 | Pelvic-femoral girdles, LL distal muscle | no | no | 130 | no |
| 38 | c.550delA/ c.1823G>A | p.(Thr184Argfs*36)/ p.(Arg608Lys) | Dystrophic pattern; immunolabelling: dysferlin deficiency; CAPN3: weak labelling on 94, 60, 30 kDa | 40 | Shoulder and pelvic-femoral girdles | 55 | Shoulder and pelvic-femoral girdles | no | no | 20 | Lumbalgia, scapula alata |
| 39 | c.550delA/ c.1981delA | p.Thr184Argfs*36/ p.Ile661* | Dystrophic pattern; immunolabelling: dysferlin deficiency; CAPN3: absence on 94, 60, 30 kDa, labelling on 45 kDa | 6 | Pelvic-femoral girdles | 7 | Pelvic-femoral girdles | no | no | 20 | Calf hypertrophy, generalised hypotonia |
| 40 | c.550delA/ **c.2245A>C** | p.(Thr184Argfs*36)/ **p.(Asn749His)** | NP | NI | NI | 63 | Shoulder and pelvic girdles | NI | NI | NI | NI |
| 41 | **c.1A>G**/c.865C>T | **p.(Met1Val)**/ p.(Arg289Trp) | Dystrophic pattern; normal immunolabelling; CAPN3: NP | NI | NI | 31 | Shoulder and pelvic-femoral girdles | NI | NI | NI | NI |
| 42 | c.133G>A/ c.133G>A | p.Ala45Thr/p.Ala45Thr | Dystrophic pattern; normal immunolabelling; CAPN3: absence on 94, 60, 30 kDa, labelling on 45 kDa | 15 | Pelvic-femoral girdles | 58 | Shoulder and pelvic-femoral girdles, back muscles, distal muscles | 55 | Achille tendons | 4 | Scapula alata, hyperlordosis, respiratory muscle weakness, pes cavus |
| 43 | c.146G>A/ c.1069C>T | p.(Arg49His)/ p.(Arg357Trp) | NP | 13 | Pelvic-femoral girdles | 16 | Pelvic-femoral girdles, LL distal muscles | no | no | 23 | no |
| 44 | **c.224A>G/ c.224A>G** | **p.Tyr75Cys/p.Tyr75Cys** | Dystrophic pattern; immunolabelling: dysferlin deficiency; CAPN3: weak labelling on 94, 60, 30 kDa | 8 | Shoulder and pelvic-femoral girdles | 33 | Shoulder and pelvic-femoral girdles, distal muscles | 30 | no | 85 | no |
| 45 | c.245C>T/ c.245C>T | p.Pro82Leu/p.Pro82Leu | Dystrophic pattern; immunolabelling: NP; CAPN3: absence on 94, 60, 30 kDa | 18 | Pelvic-femoral girdles | 35 | Shoulder and pelvic-femoral girdles | no | Achille tendons | 20 | Hyperlordosis, scapula alata |
| 46 | c.245C>T/ **c.1800+1G>A** | p.(Pro82Leu)/**splicing** | NP | 10 | Shoulder and pelvic-femoral girdles | 26 | Shoulder and pelvic-femoral girdles, distal muscles | no | no | 96 | no |
| 47 | c.245C>T/ **c.1855C>T** | p.(Pro82Leu)/ **p.(Gln619*)** | NP | 30 | Pelvic-femoral girdles | 32 | Shoulder and pelvic-femoral girdles, LL distal muscles | no | Achilles tendons | 8 | Calf hypertrophy muscle pain, scapula alata |
| 48 | c.245C>T/ c.2314_2317del | p.Pro82Leu/ p.Asp772Asnfs*3 | Dystrophic pattern; immunolabelling: dysferlin deficiency; CAPN3: absence on 94, 60, 30 kDa, labelling on 45 kDa | 13 | Pelvic-femoral girdles | 30 | Shoulder and pelvic-femoral girdles | no | Achilles tendons | 20 | Hyperlordosis, swelling of LL |
| 49 | c.509A>G/ c.509A>G | p.(Tyr170Cys)/ p.(Tyr170Cys) | NP | NI | NI | 34 | Shoulder and pelvic-femoral girdles | NI | NI | NI | NI |
| 50 | c.598_612del/ c.598_612del | p.(Phe200_Leu204del)/ p.(Phe200_Leu204del) | NP | 10 | Pelvic-femoral girdles | 15 | Shoulder and pelvic-femoral girdles, distal muscles | no | no | 86 | no |
| 51 | c.598_612del/ c.640G>A | p.(Phe200-Leu204del)/ p.(Gly214Ser) | Dystrophic and inflammatory pattern; immunolabelling: focal dysferlin deficiency; CAPN3: weak labelling on 94, 60, 30 kDa | NI | NI | 46 | Shoulder and pelvic-femoral girdles | NI | NI | NI | NI |
| 52 | c.598_612del/ **c.2245A>C** | p.Phe200-Leu204del/ **p.Asn749His** | Dystrophic pattern; immunolabelling: NP; CAPN3: absence on 94, 60, 30 kDa | 17 | Pelvic-femoral girdles | 30 | Shoulder and pelvic-femoral girdles | 24 | Achilles tendons | 85 | no |
| 53 | c.1043delG/ **c.1094G>A** | p.(Gly348Valfs*4)/ **p.(Trp365*)** | NP | NI | NI | 38 | Shoulder and pelvic-femoral girdles | NI | NI | NI | NI |
| 54 | c.1043delG/ c.1343G>A | p.(Gly348Valfs*4)/ p.(Arg448His) | Dystrophic pattern; normal immunolabelling; CAPN3: absence on 94, 60, 30 kDa, labelling on 45 kDa | 10 | Shoulder and pelvic-femoral girdles | 26 | Shoulder and pelvic-femoral girdles, abdominal muscles | 25 | Achilles tendons, pes cavus | 13 | Scapula alata |
| 55 | c.1194-9A>G/ **c.1800+1G>A** | splicing/**splicing** | NP | NI | NI | 36 | Shoulder and pelvic-femoral girdles | NI | NI | NI | NI |
| 56 | c.1194-9A>G/ c.2393C>A | splicing/p.(Ala798Glu) | Myopathic pattern, multicore/minicore pattern (congenital myopathy was also suspected); normal immunolabelling; CAPN3: NP | 24 | Shoulder and pelvic-femoral girdles | 47 | Shoulder and pelvic-femoral girdles, back muscles | no | no | 89 | Muscle pain |
| 57 | c.1250C>T/ c.1250C>T | p.(Thr417Met)/ p.(Thr417Met) | Myopathic pattern; immunolabelling: dysferlin deficiency; CAPN3: labelling on 94, 60 and 45 kDa | 9 | Pelvic-femoral girdles | 19 | Pelvic-femoral girdles, LL distal muscles | no | no | 72 | no |
| 58 | c.1322G>A/ c.1322G>A | p.Gly441Asn/ p.Gly441Asn | Dystrophic pattern; immunolabelling and WB: NP | 31 | Pelvic-femoral girdles | 48 | Shoulder and pelvic-femoral girdles | no | no | 20 | Scapula alata |
| 59 | c.1322delG/ c.2114A>G | p.(Gly441Valfs*22)/ p.(Asp705Gly) | Dystrophic and inflammatory pattern; normal immunolabelling; CAPN3: absence on 94, 60, 30 kDa, labelling on 45 kDa | 3 | Pelvic-femoral girdles | 10 | Pelvic-femoral girdles | no | Achilles tendons | 116 | Calf hypertrophy, pain of back muscles |
| 60 | c.1343G>A/ **c.2093G>A** | p.(Arg448His)/ **p.(Arg698His)** | Myopathic pattern; immunolabelling: weak dysferlin detection; CAPN3: absence on 94 and 30 kDa, labelling on 60 and 45 kDa | 47 | Pelvic-femoral girdles | 53 | Shoulder and pelvic-femoral girdles | no | no | 17 | no |
| 61 | c.1468C>T/ c.2314_2317del | p.(Arg490Trp)/ p.(Asp772Asnfs*3) | Dystrophic and inflammatory pattern; immunolabelling: focal dysferlin deficiency; CAPN3: NP | 5 | Pelvic-femoral girdles | 5 | Shoulder and pelvic-femoral girdles | no | Achilles tendons, elbows, wrists, fingers | 40 | Pain of LL muscles (after activity) |
| 62 | **c.1788_1793del**/ c.2242C>T | **p.Lys597_Lys598del**/ p.Arg748* | Myopathic pattern, selective atrophy of type I fibers; immunolabelling: dysferlin deficiency; CAPN3: NP | 18 | Shoulder geirdles | 52 | Shoulder and pelvic-femoral girdles | 50 | no | 47 | no |
| 63 | c.598_612del/ c.1746-20C>G | p.(Phe200_Leu204del)/ splicing | Dystrophic pattern ; immunolabelling: weak dysferlin detection; CAPN3: labelling on 94, 60, 30 kDa | 43 | Biceps | 61 | Shoulder girdle | no | no | NI | no |
| 64 | c.598-612del/ c.1746-20C>G | p.Phe200_Leu204del/ splicing | Dystrophic pattern; immunolabelling: dysferlin deficiency; CAPN3: absence on 94, 60, 30 kDa, labelling on 45 kDa | 25 | Pelvic-femoral girdles | 68 | Shoulder and pelvic-femoral girdles, abdominal muscles | 62 | no | NI | Asymmetry of muscle weakness on shoulder girdles |
| 65 | c.598-612del/ c.1746-20C>G | p.(Phe200_Leu204del)/ splicing | Dystrophic pattern; normal immunolabelling; CAPN3: NP | 56 | Shoulder and pelvic-femoral girdles | 66 | Shoulder and pelvic-femoral girdles, distal muscle | 65 | no | NI | no |
| 66 | c.598_612del/ c.1746-20C>G | p.(Phe200_Leu204del)/ splicing | NP | 42 | Shoulder and pelvic-femoral girdles | 46 | Shoulder and pelvic-femoral girdles | no | no | 1 | Asymmetry of muscle weakness - scapula alata on the right-hand side |
| 67 | **c.614T>C**/ c.1746-20C>G | **p.(Leu205Pro)**/splicing | Mild myopathic pattern; normal immunolabelling; CAPN3: absence on 94, 60, 30 kDa, labelling on 45 kDa | NI | NI | 49 | Shoulder and pelvic-femoral girdles | NI | NI | NI | NI |
| 68 | c.550delA/- | p.(Thr184Argfs*36)/- | NP | NI | NI | 22 | Pelvic-femoral girdles | NI | Achilles tendons, elbows | NI | NI |
| 69 | c.550delA/- | p.(Thr184Argfs*36)/- | Myopathic pattern; immunolabelling: weak dysferlin detection; CAPN3: weak labelling on 94 and 60 kDa | 2 | Shoulder girdle | 28 | Shoulder and pelvic-femoral girdles | no | no | 3 | Kyfoscoliosis, respiratory muscle weakness |
| 70 | c.598_612del/- | p.(Phe200_Leu204del)/- | NP | NI | NI | 17 | Pelvic-femoral girdles | NI | NI | NI | NI |
| 71 | 598_612del/- | p.(Phe200_Leu204del)/- | NP | 30 | Pelvic-femoral girdles | 60 | Shoulder and pelvic-femoral girdles | no | no | 9 | no |

Mutations in bold letters were detected only in Czech LGMD2A patients. NI: no information; NP: not performed; LL: lower limbs; UP: upper limbs. Immunohistochemical detection of dystrophin; dysferlin; beta-dytroglycan; alpha, beta, gamma, and delta-sarcoglycan; emerin; merosin; utrophin; and spectrin was performed.
