# Supplementary material for: Autosomal recessive limb-girdle muscular dystrophies in the Czech Republic
Source: BMC Neurol. 2014 Aug 19;14:154. doi: 10.1186/s12883-014-0154-7 (PMC4145250; doi:10.1186/s12883-014-0154-7)
Supplement: Additional file 2: Table S2. — Mutations and pathological-clinical findings identified in Czech LGMD2I, 2D, 2L, 2B, and 2E probands. [file s12883-014-0154-7-S2.docx]

Table S2. Mutations and pathological-clinical findings identified in Czech LGMD2I, 2D, 2L, 2B, and 2E probands

| **No.** | **Gen** | **Mutace (cDNA level)** | **Mutace (protein level)** | **Muscle biopsy; immunohistochemistry** | **Onset (years)** | **First manifestation of muscle weakness** | **Age (years)** | **Actual localisation of muscle weakness** | **Loss of walking (years)** | **Contra-ctures** | **CK (ukat/l)** | **Other symptoms** |
| --- | --- | --- | --- | --- | --- | --- | --- | --- | --- | --- | --- | --- |
| 72 | *FKRP* | c.826C>A/  c.826C>A | p.(Leu276Ile)/ p.(Leu276Ile) | Dystrophic pattern; immunolabelling: alpha-dystroglycan deficiency, dysferlin deficiency on 30% of MF | 21 | Pelvic-femoral girdles | 45 | Pelvic-femoral girdles | no | no | 92 | no |
| 73 | *FKRP* | c.826C>A/ c.826C>A | p.(Leu276Ile)/ p.(Leu276Ile) | Dystrophic pattern; normal immunolabelling | 18 | Pelvic-femoral girdles | 45 | Shoulder and pelvic-femoral girdles, trunk muscles | Weehlchair bound for longer distance | no | 23 | Hyperlordosis, pain of spine, respiratory muscle weakness, dilated cardiomyopathy, swelling of LL |
| 74 | *FKRP* | c.826C>A/ c.826C>A | p.(Leu276Ile)/ p.(Leu276Ile) | NP | NI | NI | 30 | Shoulder and pelvic-femoral girdles | NI | NI | NI | NI |
| 75 | *FKRP* | c.826C>A/ c.826C>A | p.(Leu276Ile)/ p.(Leu276Ile) | Dystrophic pattern; normal immunolabelling | 10 | Pelvic-femoral girdles | 28 | Shoulder and pelvic-femoral girdles, abdominal muscles, distal UL muscles | no | no | 11 | Scoliosis, respiratory muscle weakness, disturbance of independent locomotion and stability |
| 76 | *FKRP* | c.826C>A/ c.826C>A | p.(Leu276Ile)/ p.(Leu276Ile) | NP | 22 | Shoulder and pelvic-femoral girdles | 27 | Shoulder and pelvic-femoral girdles | no | no | 21 | Calf hypertrophy |
| 77 | *FKRP* | c.826C>A/ c.826C>A | p.(Leu276Ile)/ p.(Leu276Ile) | Dystrophic pattern; normal immunolabelling | 12 | Pelvic-femoral girdles | 20 | Pelvic-femoral girdles | no | no | 65 | no |
| 78 | *FKRP* | c.826C>A/ c.826C>A | p.(Leu276Ile)/ p.(Leu276Ile) | Dystrophic pattern; immunolabelling: alpha-dystroglycan deficiency (smal groups of alpha-dystroglycan positive MF) | 13 | Pelvic-femoral girdles | 27 | Pelvic-femoral girdles | no | no | 72 | no |
| 79 | *FKRP* | c.826C>A/ c.947C>G | p.(Leu276Ile)/ p.(Pro316Arg) | NP | 5 | Pelvic-femoral girdles | 8 | Pelvic-femoral girdles | no | Achilles tendons | 65 | Calf hypertrophy, pain of LL muscles (associated with fatigue) |
| 80 | *FKRP* | 826C>A/ **c.1076G>C** | p.(Leu276Ile)/ **p.(Trp359Ser)** | Dystrophic pattern; normal immunolabelling | 4 | Pelvic-femoral girdles | 25 | Shoulder and pelvic-femoral girdles, abdominal muscles | Weehlchair bound for longer distance | Achilles tendons | 164 | Calf hypertrophy, cardiomyopathy |
| 81 | *SGCA* | c.229C>T/ c.229C>T | p.(Arg77Cys)/ p.(Arg77Cys) | NP | 8 | Pelvic-femoral girdles | 44 | Shoulder and pelvic-femoral girdles, trunk muscles, distal muscles | 17 | Achilles tendons | 4 | Scoliosis, respiratory muscle weakness |
| 82 | *SGCA* | c.157+1G>A/ c.850C>T | splicing/ p.(Arg284Cys) | Dystrophic pattern; immunolabelling: absence or weak labelling of alpha-sarcoglycan on 80% of MF, absence or weak labelling of beta-sarcoglycan on 20% of MF, absence or weak labelling of gamma-sarcoglycan on 10% of MF, weak labelling of alpha-dystroglykan on 10% of MF, absence of dysferlin on 5% of MF | 8 | Pelvic-femoral girdles | 15 | Pelvic-femoral girdles | no | no | 96 | no |
| 83 | *SGCA* | c.157+1G>A/ c.850C>T | splicing/ p.(Arg284Cys) | NP | 6 | Pelvic-femoral girdles | 18 | Shoulder and pelvic-femoral girdles | no | no | 68 | no |
| 84 | *SGCA* | c.229C>T/c.739G>A | p.(Arg77Cys)/ p.(Val247Met) | Dystrophic pattern; normal immunolabelling | 15 | Shoulder and pelvic-femoral girdles | 39 | Shoulder and pelvic-femoral girdles, trunk muscles | no | no | 20 | Muscle cramps |
| 85 | *SGCA* | c.290A>G**/ c.303dupA** | p.(Asp97Gly)/ **p.(Gln101Glnfs*4)** | Dystrophic pattern; immunolabelling: absence of alpha, beta, gamma, delta-sarcoglycans, weak labelling of alpha, beta-dystroglycans , and dysferlin | 6 | Pelvic-femoral girdles | 10 | Pelvic-femoral girdles | no | Achilles tendons | 39 | Hyperlordosis, calf hypertrophy |
| 86 | *SGCA* | c.229C>T/ c.308T>C | p.(Arg77Cys)/  p .(Ile103Thr) | NP | NI | NI | 10 | Pelvic-femoral girdles | NI | NI | NI | NI |
| 87 | *ANO5* | c.191dupA/ *c. 966A>T* | p.(Asn64Lysfs*15)/ *p.(Leu322Phe)* | Myopathic pattern, normal immunolabelling | 12 | Pelvic-femoral girdles | 33 | Pelvic-femoral girdles | no | no | 96 | no |
| 88 | *ANO5* | c.191dupA/ c.2272C>T | p.(Asn64Lysfs*15)/ p.(Arg758Cys) | Fat and fibrous tissue | 45 | Shoulder and pelvic-femoral girdles | 49 | Shoulder and pelvic-femoral girdles, trunk muscles | no | no |  | Hyperlordosis |
| 89 | *ANO5* | c.191dupA/ c.2272C>T | p.(Asn64Lysfs*15)/ p.(Arg758Cys) | Mild dystrophic pattern; normal immunolabelling | NI | NI | 28 | Shoulder and pelvic-femoral girdles | NI | NI | NI | NI |
| 90 | *DYSF* | **c.3832C>T/ c.5509G>T** | **p.(Gln1278*)**/ **p.(Asp1837Tyr)** | NP | 22 (hyperCKemia from 14 year of age) | Pelvic-femoral girdles | 34 | Pelvic-femoral girdles, trunk muscles, distal LL muscles | no | no | 68 | no |
| 91 | *DYSF* | c.509C>A/  c.610C>T/ c.1120G>C/ **c.5907G>C** | p.(Ala170Glu)/ p.(Arg204*)/ p.(Val374Leu)/ **p.(Trp1969Cys)** | Mild dystrophic pattern immunolabelling: dysferlin deficiency | 34 | Pelvic-femoral girdles | 39 | Pelvic-femoral girdles, distal LL muscles, paravertebral muscles | no | no | 82 | Mild asymmentry of muscle weakness, muscle pain |
| 92 | *SGCB* | c.341C>T/ c.341C>T | p.(Ser114Phe)/ p.(Ser114Phe) | Dystrophic and inflammatory pattern; normal immunolabelling | 6 | Pelvic-femoral girdles | 15 | Pelvic-femoral girdles | no | no | 100 | Calf hypertrophy |

Mutations in bold letters were detected only in Czech LGMD2 patients. NI: no information; NP: not performed; LL: lower limbs; MF: muscle fibres. Immunohistochemical detection of dystrophin; dysferlin; beta-dytroglycan; alpha, beta, gamma, and delta-sarcoglycan; emerin; merosin; utrophin; and spectrin was performed, and also alpha-dystroglycan in patients 72, 78, 82, 85. The variant c.966A>T written in italics is probably a nucleotide polymorphism (LMDP, dbSNP-rs7481951).
