# Supplementary material for: Autosomal recessive limb-girdle muscular dystrophies in the Czech Republic
Source: BMC Neurol. 2014 Aug 19;14:154. doi: 10.1186/s12883-014-0154-7 (PMC4145250; doi:10.1186/s12883-014-0154-7)
Supplement: Additional file 3: Table S3. — In silico prediction of effects of selected missense mutations. [file s12883-014-0154-7-S3.docx]

Table S3. *In silco* prediction of effects of selected missense mutations

| Gene | Mutation | PolyPhen-2 HumVar (score) | SIFT (score) | PON-P (probability of pathogenicity) |
| --- | --- | --- | --- | --- |
| *CAPN3* | p.Tyr75Cys | possibly damaging (0.823) | affect protein function (0.00) | neutral (0.15) |
| *CAPN3* | p.(Leu205Pro) | probably damaging (1.000) | affect protein function (0.00) | unclassified (0.69) |
| *CAPN3* | p.(Leu484Pro) | probably damaging (1.000) | affect protein function (0.00) | unclassfied (0.36) |
| *CAPN3* | p.(Arg698His) | probably damaging (0.999) | tolerated (0.09) | pathogenic (0.99) |
| *CAPN3* | p.(Asn749His) | probably damaging (1.000) | tolerated (0.38) | unclassified (0.19) |
| *FKRP* | p.(Trp359Ser) | probably damaging (0.999) | affect protein function (0.00) | unclassified (0.37) |
| *ANO5* | p.(Leu322Phe) | benign (0.022) | tolerated (0.23) | unclassified (0.27) |
| *DYSF* | p.(Ala170Glu) | benign(0.017) | tolerated (1.0) | neutral (0.02) |
| *DYSF* | p.(Val374Leu) | benign (0.009) | tolerated (0.26) | neutral (0.13) |
| *DYSF* | p.(Asp1837Tyr) | probably damaging (0.999) | affect protein function (0.00) | pathogenic (0.97) |
| *DYSF* | p.(Trp1969Cys) | probably damaging (0.999) | affect protein function (0.00) | unclassified (0.28) |
